# Phylogenetic Assessment of *Gazella bennettii*: A Genetic Framework for the Conservation of the Endangered Jebeer in Iran

**DOI:** 10.1002/ece3.70954

**Published:** 2025-02-12

**Authors:** Davoud Fadakar, Mansoureh Malekian, Mahmoud Reza Hemami, Hamid Reza Rezaei, Hannes Lerp, Eva V. Bärmann

**Affiliations:** ^1^ Department of Natural Resources Isfahan University of Technology Isfahan Isfahan Iran; ^2^ Department of Fishery and Environment Gorgan University of Agricultural Science and Natural Resources Gorgan Golestan Iran; ^3^ Natural History Collections Museum Wiesbaden Wiesbaden Germany; ^4^ Zoological Research Museum Alexander Koenig Bonn Germany

**Keywords:** conservation genetics, desert ungulate, India, Indus River, vicariance

## Abstract

The chinkara ( *Gazella bennettii* , Sykes 1831) exhibits a broad distribution from Iran to India and has been categorized into five species: *G. bennettii* , *G. christyi* , and *G. salinarum* representing the Indian chinkara, and *G. fuscifrons* and *G. shikarii* pertaining to the Iranian chinkara (jebeer). This classification within the “*Gazelle bennettii* group” is solely based on morphological data, lacking genetic information. To investigate the potential presence of multiple species within the group and to determine subspecific variations, we sampled jebeer in Iran and conducted phylogenetic analyses using cytochrome *b*, COI, and sequences from two nuclear introns (CHD2 and ZNF618). Our mitochondrial data revealed a significant divergence within the “*Gazelle bennettii* group,” identifying two lineages: the Iranian lineage (jebeer) and the Indian lineage (chinkara). Estimates of divergence time suggest that the split between jebeer and chinkara occurred approximately 0.895 million years ago, possibly associated with a vicariant event caused by the Indus River. These findings have important implications for assessing species conservation statuses on the IUCN Red List because an endangered lineage (jebeer) is currently grouped together with a non‐threatened one (chinkara) under the same global assessment, which underestimates the true endangered status of jebeer. In Iran, the haplotype distribution map of the jebeer can serve as a fundamental genetic framework guiding conservation efforts across Iranian protected areas and captive breeding centers. Therefore, we recommend any future management plan should be based on these findings and treat these two lineages separately.

## Graphical abstract

Our molecular genetic analyses provide resolution for the controversial status of jebeer and chinkara, providing a basis for future assessments by the IUCN and national authorities in Iran. Furthermore, these data enable us to make informed decisions regarding crucial areas of jebeer conservation.

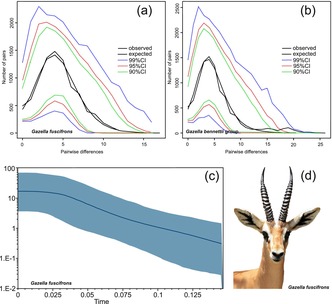

## 1 Introduction

Conservation biology primarily aims to preserve genetic diversity in order to maintain the evolutionary potential of species (Crandall et al. 2000). Phylogenetics, which uses genetic data to determine evolutionary relationships among populations and species, also makes significant contributions to conservation efforts (Moritz 1995). Phylogeny, along with its application in phylogeography, provides valuable insights into how current genetic patterns have evolved over time. Phylogenetic analysis aids species conservation by clarifying taxonomic status, identifying unique evolutionary lineages, determining relictual and recently derived species, and establishing phylogenetic value for setting conservation priorities (Sandall et al. 2023). Phylogenetic studies conducted below the species level are valuable for identifying distinct genetic lineages with unique evolutionary histories, which can serve as evolutionary significant units (Hoelzel 2023). This is particularly important for species complexes, such as the chinkara ( *G. bennettii* ), where genetic and morphological variations have not been clearly defined.

The Iranian chinkara and the goitered gazelle ( *G. subgutturosa* ) represent two widely dispersed gazelle species across the Iranian Plateau. Goitered gazelles primarily prefer open plains, inhabiting steppes and semi‐desert plains (Karami et al. 2002; Fadakar et al. 2013; Fadakar, Bärmann, et al. 2020; Hemami et al. 2020). In contrast, Iranian chinkaras thrive in arid desert habitats with limited water access, particularly in central Iranian regions such as Dasht‐e Lut and Dasht‐e Kavir (Akbari 2014). The chinkara's distribution ranges from the northern part of the central deserts of Iran to the south and southeastern regions, as well as southwestern Afghanistan, Pakistan, and central and western India. Lerp et al. (2016b) suggested that the Middle East is the likely origin of the genus *Gazella*. Fadakar, Malekian, et al. (2020) further affirmed the Iranian origin of goitered gazelles through mitochondrial DNA (mtDNA) analysis, revealing the existence of two subspecies in Iran.

**FIGURE 1:**
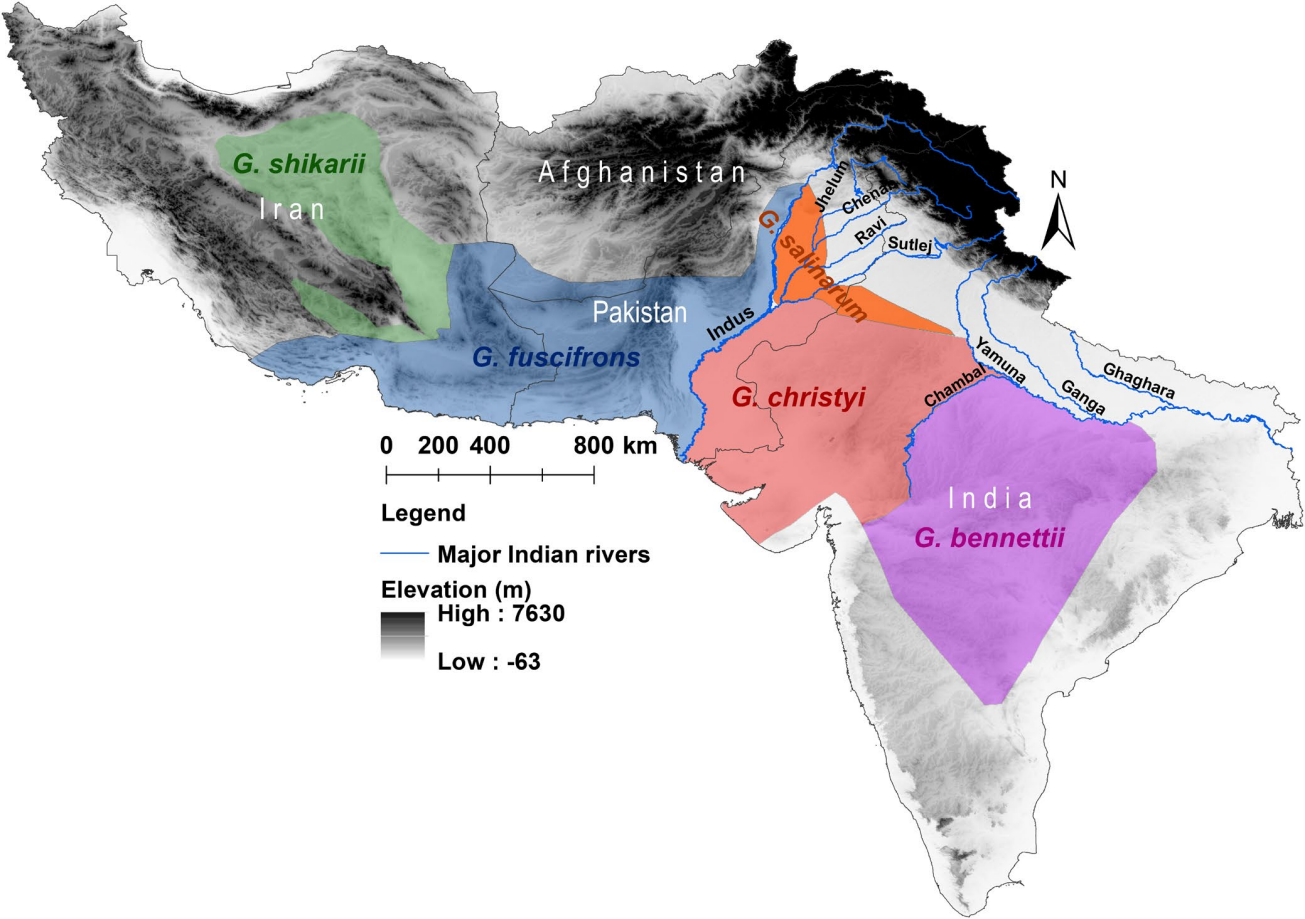
Distribution of the *Gazella bennettii* group: *Gazella bennettii* , in Violet; *G. salinarum*, in fire red; *G. christyi* , in red; *G. shikarii*, in green; and *G. fuscifrons*, in blue. Polygons correspond to the distribution of suggested (sub)species for the *G. bennettii* group based on the morphometric analyses (Groves and Grubb 2011). Modified from Rahmani (1990) and Akbari (2014). The background hillshade was made using the Shuttle Radar Topography Mission (SRTM) elevation model (http://srtm.csi.cgiar.org) in QGIS v.3.10; country boundaries were downloaded from the DIVA‐GIS dataset (http://www.diva‐gis.org/Data), and the layout was made in QGIS v.3.10.

For the taxonomic classification of the *Gazella bennettii* group, subspecies delineation primarily relies on morphological data in Iran (Hemami and Groves 2001). Groves (1993) used discriminant analysis on male *G. bennettii* skulls and identified three subspecies in Iran: *G. b. fuscifrons* (Blanford, 1873) from the southern and southeastern coastal plains and deserts, *G. b. shikarii* (Groves 1993) from central deserts to northern habitats, and *G. b. karamii* (Groves 1993) from Borazjan near Bushehr in southwestern Iran. Notably, Groves observed that these subspecies do not cluster with Indian chinkara specimens (Karami et al. 2002). In the case of *G. b. karmaii*, the skull (ZMB_MAM_41400) was identified as a *G. marica* based on morphometric data (Bärmann et al. 2013), and cytochrome *b* (cyt *b*) sequences have confirmed the existence of *G. marica* in southwestern Iran (Fadakar et al. 2019). Later, Groves (2003) introduced three new subspecies from India, namely *G. b. bennettii* (Sykes, 1831), *G. b. christyi* (Blyth, 1842), and *G. b. salinarum* (Groves 2003). In their most recent systematic analysis, Groves and Grubb (2011) utilized discriminant analysis with nine variables and elevated some of the previously known subspecies to the species level, identifying five species within the “ *Gazella bennettii* group” (hereafter referred to as the species complex *Gazella bennettii* group) (Figure 1). Two of these species (*G. fuscifrons* from southeastern to southern Iran and *G. shikarii* from central to northern parts of Iran) are associated with the Iranian chinkara (referred to as jebeer hereafter). The other three species ( *G. bennettii* , *G. christyi* , and *G. salinarum*) correspond to the Indian chinkara (referred to as chinkara hereafter). As indicated by Heller et al. (2013), the increase in species number observed in the taxonomic revision by Groves and Grubb (2011) exemplifies taxonomic inflation (Isaac et al. 2004). Moreover, the elevation of subspecies to full species rank within the *Gazella bennettii* group, without genetic substantiation, highlights a misuse of the phylogenetic species concept (Zachos et al. 2013). This fact underlines the necessity for carrying out a genetic study on the species complex.

From a conservation perspective, the *G. bennettii* group, including all populations across its entire known range, is currently considered a single species by the IUCN (IUCN SSC Antelope Specialist Group 2017). If it is determined that the Iranian populations of the species comprise a distinct clade from the Indian ones, an endangered taxon (jebeer) (Yusefi et al. 2019) may be currently grouped with a non‐threatened one (chinkara) (IUCN SSC Antelope Specialist Group 2017), potentially leading to inappropriate translocation or captive breeding decisions (Zachos et al. 2013). Conservation efforts require a genetic study of *G. bennettii* , to determine if Iranian and Indian populations belong to the same species and to clarify subspecific variations. The primary goals of this study were to (i) investigate the potential presence of multiple species within the *G. bennettii* group and (ii) determine the subspecific variations, providing a genetic framework for the conservation of jebeer populations in Iran.

## 2 Materials and Methods

### 2.1 Distribution Range and Sampling

The IUCN map of *G. bennettii* (IUCN SSC Antelope Specialist Group 2017) has been updated to reflect the distribution of the *G. bennettii* group, incorporating descriptions from previous studies (Rahmani 1990; Hemami and Groves 2001; Akbari 2014; Mirzakhah et al. 2015). Figure 1 shows the polygons for each species suggested by Groves and Grubb (2011), as well as the positions of major rivers in Pakistan and India.

**FIGURE 2:**
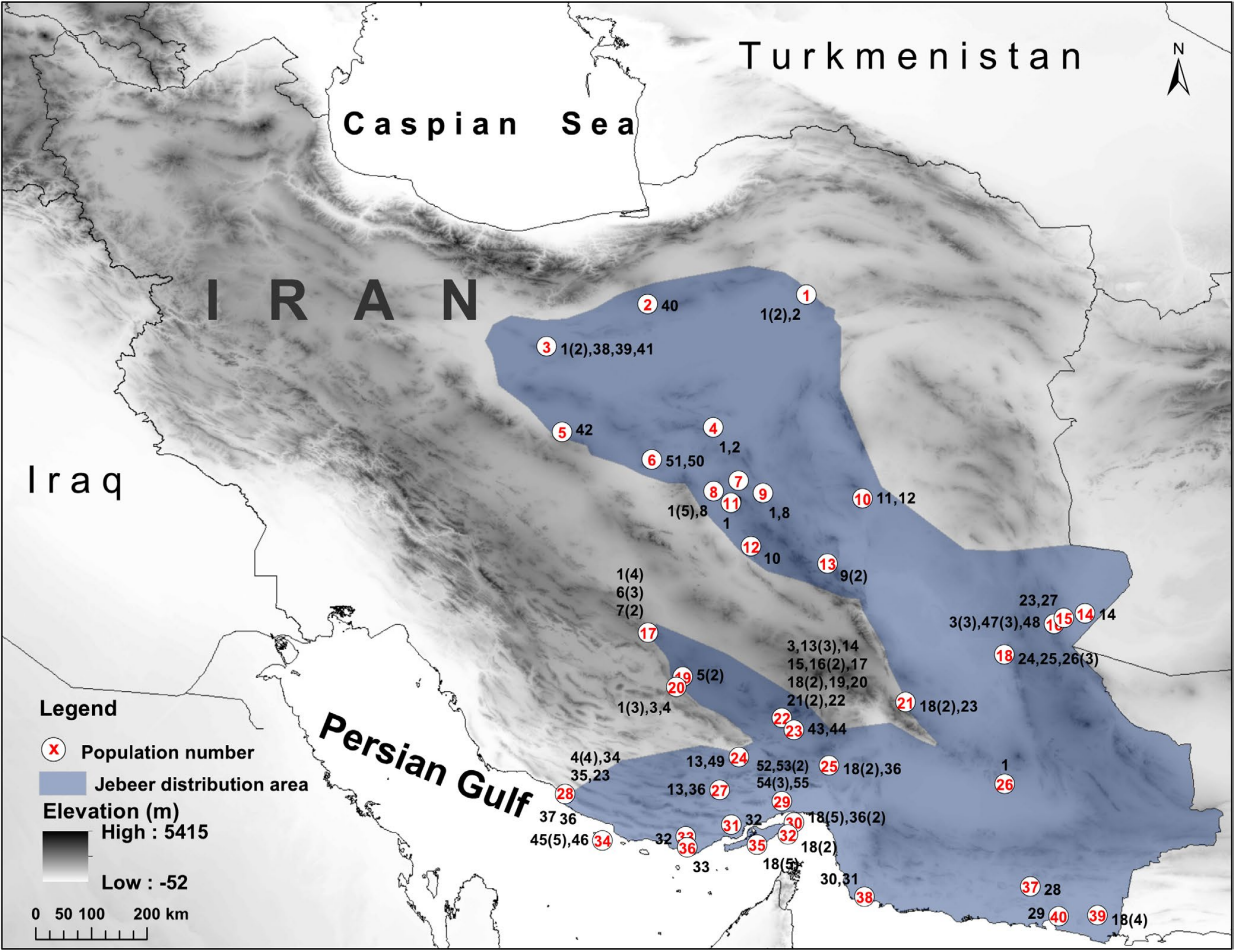
Distribution map of haplotypes of jebeer in Iran. Populations are sorted based on the latitude from north to south (in red), and identified haplotypes and frequency are shown for each population (in black). Populations codes are: 1 = Turan National Park (NP), 2 = Chah Shirin No‐Hunting Area (NHA), 3 = Kavir NP, 4 = Abbas Abad Wildlife Refuge (WR), 5 = Ardestan, 6 = Siahkoh NP, 7 = Saqand Desert, 8 = Darre Anjir WR, 9 = Bahabad NHA, 10 = Naybandan WR, 11 = Ariz WR, 12 = Bafq Protected Area (PA), 13 = Ravar, 14 = Shile PA, 15 = Rahmatzei Breeding Center, 16 = Mokesorkh NHA, 17 = Boruyeh WR, 18 = Bolbolab NHA, 19 = Bahram‐e Goor PA, 20 = Qatruyeh NP, 21 = Sang‐e Mes PA, 22 = Khabr NP, 23 = Kerman, 24 = Tarom PA, 25 = Mosafer Abad Plain, 26 = Bazman NHA, 27 = Hormoud PA, 28 = Nayband NP, 29 = Geno PA, 30 = Hormoz Island, 31 = Bandar‐e Khamir, 32 = Larak Island, 33 = Barkohi Village, 34 = Lavan Island, 35 = Hengam Island, 36 = Bandar‐e Lengeh, 37 = Koh‐e Pozak PA, 38 = Sohran Village, 39 = Gando PA, and 40 = Chabahar.

In total, 134 fecal and tissue samples of jebeer were collected throughout Iran (Figure 2). The locality information, species, and the type of material are summarized in Table S1. Tissue samples were collected from dried heads and legs of confiscated animals from illegal hunting, as well as animals that died from natural causes in the protected areas of Iran. Jebeer and goitered gazelles co‐occur in certain regions of central Iran; therefore, it is essential to distinguish between the two species before collecting fresh samples (Fadakar, Bärmann, et al. 2020). The jebeer is smaller than the goitered gazelle, and both sexes of the jebeer possess straight horns. In contrast, male goitered gazelles have distinctly curved horns, while females do not have horns. All fresh fecal samples were collected noninvasively and stored in 96% ethanol. This study was conducted with permission (95/28426) from the Iranian Department of Environment (DoE), which authorized access to all sampling locations.

### 2.2 DNA Extraction, Amplification, and Sequencing

Whole genomic DNA was extracted from samples using the AccuPrep Genomic DNA Extraction Kit (Bioneer) following the manufacturer's instructions. Polymerase chain reaction (PCR) was performed to amplify the entire coding region of the cyt *b* gene of mtDNA using CYTB_F (5′‐CCCCACAAAACCTATCACAAA‐3′) and CYTB_R (5′‐AGGGAGGTTGGTTGTTCTCC‐3′) primers (Pedrosa et al. 2005; Rezaei et al. 2010).

The reaction mixture was prepared in a 25 μL volume, containing 1 unit of Euro Taq DNA polymerase, 10 μM Tris–HCl, 30 μM KCl, 1.5 mM MgCl_2_, 250 μM of each dNTP, and 2 pmol of primers (Bioneer, South Korea). For the thermocycling of CYTB_F and CYTB_R primers, we followed the protocol (Rezaei et al. 2010): 10 min at 95°C, followed by 35 cycles of 30 s at 95°C, 30 s at 55°C, and 60 s at 72°C, and finally followed by 7 min at 72°C. Sanger sequencing was performed using the BigDye Terminator Cycle Sequencing Kit v.3.1 (Applied BioSystems), and electrophoresis of the purified sequencing product was carried out on an ABI PRISM 3730xl automatic sequencer.

Chromodomain–helicase–DNA‐binding protein 2 (CHD2) and zinc finger protein 618 (ZNF618) are two nuclear introns for *Gazella* (Lerp et al. 2016a) that can differentiate between *G. subgutturosa* and *G. bennettii* due to limited variation and high consistency in the species delineation (Fadakar, Malekian, et al. 2020). These two introns were selected to amplify jebeer samples from Iran using the primers from Lerp et al. (2016b). The PCR was conducted in a GeneAmp 2720 Thermo Cycler (Applied Biosystems) using the QIAGEN Multiplex PCR Kit in a 20 μL volume. The mixture included 2 μL of Q‐Solution, 10 μL of QIAGEN Multiplex PCR Master Mix (including HotStarTaq DNA Polymerase, QIAGEN Multiplex PCR Buffer, and dNTP Mix), and 1.6 μL of each primer (10 pmol/μL). The protocol involved an initial step of 15 min at 95°C (initial step) followed by 38 cycles of 35 s at 95°C, 60 s at 60°C, 60 s at 72°C, and a final elongation step of 10 min at 72°C. PCR products were purified using 6 μL of HT ExoSAP‐IT (Thermo Scientific). Purified PCR products were sent to Macrogen for Sanger sequencing. Sequences were edited for correction using SeqScape v.2.6 software (Applied Biosystems), and all new sequences have been submitted to GenBank (PQ809265‐PQ809448, Table S1). Sequences were aligned using the Clustal W algorithm (Thompson et al. 1994) implemented in MEGA v.5 (Tamura et al. 2011).

### 2.3 Phylogenetic Analyses

#### 2.3.1 Cyt *b* Trees

For the phylogenetic analysis of the cyt *b* dataset, only one representative for each haplotype was used. We added 15 previously published *G. bennettii* sequences from GenBank, including five sequences (two from Iran, one from Pakistan, and two with an unknown origin) from captive individuals at King Khalid Wildlife Research Center (KKWR) in Saudi Arabia, and two Pakistani sequences from Al Wabra Wildlife Preservation (AWWP) in Qatar. Also, we included eight shorter sequences (415 bp) from free‐ranging animals in India (Table S1).

The best model of nucleotide substitution (HKY + G) was selected based on Bayesian Information Criterion (BIC) scores using jModelTest v.0.1.1 (Posada 2008) for unpartitioned analysis.

Also, the best‐fitting partitioning scheme and nucleotide substitution models were estimated using a greedy search algorithm by PhyML (Guindon et al. 2010) in PartitionFinder v.2.1.1 (Lanfear et al. 2012, 2017). We tested partitioning schemes that involved dividing protein‐coding genes into 1st, 2nd, and 3rd codon positions. Models were selected based on BIC values. We found that the optimal partitioning scheme includes three partitions (optimal models are indicated in brackets): 1st codon (SYM + G), 2nd codon (HKY + I), and 3rd codon (GTR + I).

Bayesian inference analyses (unpartitioned and partitioned) were conducted using MrBayes v.3.2 (Ronquist et al. 2012) with two independent runs of four Markov chains (one cold and three heated) over 10,000,000 generations, with samples taken every 1000 generations. The first 25% of the sampled trees and estimated parameters were discarded as burn‐in. Convergence of the model parameters was monitored using the program Tracer v.1.7.1 (Rambaut et al. 2018). The consensus phylogenetic trees were then edited using FigTree v.1.4.4 (http://tree.bio.ed.ac.uk/software/figtree/).

#### 2.3.2 Cyt *b* and Two Nuclear Introns Tree

Based on the alignment conducted by Lerp et al. (2016a), we generated a concatenated alignment of all three markers, namely cyt *b* and two nuclear introns (ZNF618 and CHD2), by incorporating all sequences from the *Gazella bennettii* group into the sequences originally provided by Lerp et al. (2016a) for each marker. The final alignment consists of 2506 bp, and phylogenetic analysis of the concatenated dataset of cyt *b* and two nuclear introns was carried out similarly to the analysis of cyt *b* trees (as described above). The optimal partitioning scheme included three partitions: the 1st codon of cyt *b* and ZNF618 (HKY + I), the 2nd codon of cyt *b* and CHD2 (HKY + I), and the 3rd codon of cyt *b* (GTR + G).

#### 2.3.3 COI Tree

We utilized mitochondrial cytochrome *c* oxidase I (COI) gene sequences (1545 bp) from Pakistan and India to specifically examine samples from the eastern and western regions of the Indus River. These sequences were aligned to NC020703 and JN632635 (H56 in the cyt *b* dataset) using the Clustal W algorithm (Thompson et al. 1994) implemented in MEGA v.5 (Tamura et al. 2011). Phylogenetic analysis of the COI dataset was conducted in a similar manner to the analysis of cyt *b* trees (as described above), utilizing an optimal partitioning scheme that included three partitions: 1st codon (SYM + I + G), 2nd codon (F81), and 3rd codon (GTR + G).

#### 2.3.4 Molecular Clock

The BEAST v.2.6.6 program package (Bouckaert et al. 2019) was used to co‐estimate the topology and divergence times based on the cyt *b* dataset. We used the BEAUti program to set up the MCMC run with the following parameters: the partitioning scheme and corresponding model (HKY + G) as indicated by PartitionFinder v.2.1.1 (Lanfear et al. 2012, 2017), linked trees enabled, uncorrelated relaxed clock, and the tree prior specified as the Yule process of speciation. The MCMC analyses were run for 30 million generations, with sampling every 3000 generations. Although Antilopini and Caprini have rich fossil records, phylogenetic relationships have not yet been clarified (Bibi 2013). Therefore, we used normally distributed priors with a mean of 0.015 substitutions per Mya for the cyt *b* gene (Ho et al. 2005) and standard deviations of 0.005–0.025 for 95% confidence intervals (Lerp et al. 2011). Analysis of the posterior distributions of tree likelihood and other parameters using Tracer v.1.7.1 (Rambaut et al. 2018) showed effective sample size (ESS) values > 200. TreeAnnotator v.2.6.6 was then used to discard the first 25% of trees as burn‐in and extract the maximum clade credibility tree with nodes scaled to the median height obtained from the posterior sample. The visualization was done using FigTree v.1.4.4 (http://tree.bio.ed.ac.uk/software/figtree/).

### 2.4 Haplotype Networks

A median‐joining (MJ) network was constructed for 141 cyt *b* sequences, which included 118 new complete sequences from Iran. The dataset also comprised 16 sequences (MT811623‐MT811638) from Fadakar, Bärmann, et al. (2020), and 7 previously published sequences from GenBank. The software PopART v.1.7 (Leigh and Bryant 2015) was used with default settings to create the network. Latitude and longitude were then used to cluster Iranian populations into seven groups, excluding the islands in the Persian Gulf (Hormoz, Hengam, Larak, and Lavan) and Nayband National Park (NP), a breeding center with translocated individuals from various source populations. Sequences from GenBank were categorized into three groups: Pakistan, KKWR, and AWWP.

### 2.5 Population Differentiation and Expansion

Cyt *b* sequence polymorphism indices and diversity values, such as the number of haplotypes (*H*), polymorphic (segregating) sites (*S*), haplotype diversity (*h*), nucleotide diversity (*π*), and the mean number of pairwise differences within a group (*k*), were estimated using DnaSP v.5 (Librado and Rozas 2009). This analysis was conducted for the *G. bennettii* group as a whole, including the species we propose, namely *G. fuscifrons* [found in Iran and Pakistan (JN410341 and KU560634 from KKWR) (presumably west of the Indus River)], and *G. bennettii* [found in India and Pakistan (JN632635 and NC020703 from AWWP, and KU560633, JN410340, JN410357 from KKWR) (presumably east of the Indus River)].

We further estimated mismatch distributions of cyt *b* dataset separately for the *G. bennettii* group and the putative species *G. fuscifrons* from Iran (as mentioned earlier) to test if their frequency graph shows a chaotic/multimodal pattern characteristic of populations in demographic equilibrium, or a unimodal profile, which is found in populations that have experienced recent geographic expansion (Hey and Nielsen 2004). The test was performed in Arlequin v.3.5.2.2 (Excoffier and Lischer 2010) under the null hypothesis that the observed data fit the sudden expansion model, using a generalized least squares approach with 1000 bootstrap replicates. Other statistics for analyzing population expansions or declines were also calculated using DnaSP v.5 (Librado and Rozas 2009), such as *R*_2_ (Ramos‐Onsins and Rozas 2002), which utilizes information on the frequency of segregating sites, and Fu's *F*_s_ (Fu 1997), where a negative value indicates recent demographic expansion.

In addition, a coalescent‐based Bayesian Skyline Plot (BSP) (Drummond et al. 2005) of cyt *b* dataset was reconstructed using the BEAST v.2.6.6 program package (Bouckaert et al. 2019) with the HKY + G substitution model and empirical base frequencies, running 10^7^ MCMC iterations. A strict molecular clock model (0.015 substitutions per Mya for the cyt *b* gene (Ho et al. 2005; Lerp et al. 2011)) was used to achieve ESS larger than 200. Convergence was visually checked, and the plot was visualized in Tracer v.1.7.1 (Rambaut et al. 2018).

## 3 Results

The extraction and amplification of mitochondrial DNA sequences from fecal samples were successful, resulting in 118 new cyt *b* sequences from Iran. We also obtained new CHD2 sequences (669 bp) for 35 specimens and 31 new sequences of ZNF618 (689 bp) from jebeer populations in Iran (see Table S1). No results were obtained from 28 tissue samples, presumably due to improper storage conditions.

### 3.1 Phylogenetic Trees

**FIGURE 3:**
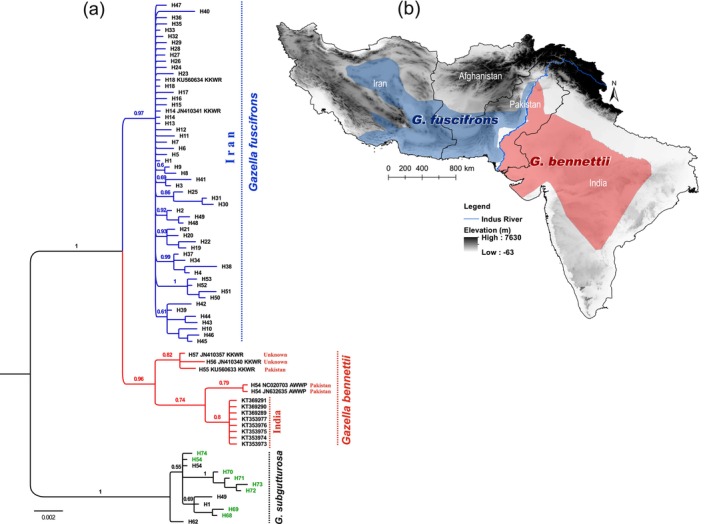
Phylogenetic tree and distribution of *G. fuscifrons* and *G. bennettii*. (a) Phylogeny of jebeer (blue), chinkara (red), and goitered gazelle × jebeer hybrids (green) from Bayesian analysis of cyt *b* gene sequences. The trees were summarized with the majority‐rule consensus tree. Numbers above branches are posterior probabilities. (b) Distribution of *G. fuscifrons* (west of the Indus River) and *G. bennettii* (east of the Indus River) based on the phylogenetic trees.

The phylogenetic analysis of complete cyt *b* sequences resolves the *G. bennettii* group (*G. fuscifrons* and *G. bennettii* ) as sister species to *G. subgutturosa* (Figure 3a). In this tree, all jebeer samples from Iran, as well as KU560634 and JN410341 from KKWR (originating from Iran based on the KKWR data), form a distinct clade (jebeer clade) with strong nodal support (posterior probability [PP] = 1), separate from chinkara sequences from India, Pakistan, and two sequences of unknown origin (chinkara clade). The chinkara clade comprises a paraphyletic group (PP = 0.96) of sequences from Pakistan and other captive individuals, along with a separate subclade of sequences from India (PP = 0.8) (Figure 3a).

The trees resulting from the partitioning scheme of cyt *b* dataset (Figure S1a) and the concatenated analysis of cyt *b* and the two nuclear introns dataset (Figure S1b) were similar to the single‐gene tree of cyt *b* dataset without partitioning (Figure 3a). This similarity occurred because the two selected nuclear markers do not exhibit sufficient variation (Tables S2 and S3), causing the concatenated tree to primarily reflect the signal from the cyt *b* marker. In all trees, *G. fuscifrons* appears as a sister species to *G. bennettii*.

Sequences from the western and eastern regions of the Indus River were found in two separate clades of COI sequences (PP = 1) (Figure S2). Divergence times estimated using the cyt *b* dataset indicate that the jebeer and chinkara clades diverged approximately 0.895 Mya (95% highest posterior density [HPD] = 0.34–1.96 Mya) during the Pleistocene (Figure S3). The divergence between the *G. bennettii* group and *G. subgutturosa* was estimated at 1.18 Mya (95% HPD = 0.51–2.55 Mya).

For ZNF618 (Table S2), jebeer and chinkara sequences share an insertion/duplication of 6 bp after position 212, which is absent in other gazelle species. The absence of this of 6 bp insertion/duplication was also observed in 11 jebeer specimens and two chinkara specimens (see Table S2 for other variation sites). For CHD2 (Table S3), only four jebeer specimens show variation sites, including BKLT3 (“T” at positions 109 and 216), BGOR3 and HRMD5 (“C” at position 221), and a jebeer specimen from KKWR (ID = 9) (“Y” at positions 236 and 273). All other CHD2 sequences show no variation between jebeer and chinkara specimens.

### 3.2 Haplotype Networks

The reconstructed MJ network, based on the 1140 bp fragment of cyt *b*, provides an overview of the haplotype distribution and relationships within the *G. bennettii* group (Figure S4). In total, 57 haplotypes were identified in the MJ network, including 53 haplotypes from Iran (H1‐H53), H54 (JN632635 and NC020703) and H55 (KU560633) from Pakistan, and H56 (JN410340) and H57 (JN410357) from KKWR of unknown origin.

In Iran, all haplotypes are closely connected, mostly separated by only one or two mutations, except for five mutations between H33 and H40, four mutations between H25 and H31, H1 and H52, and H4 and H38, and three mutations between H39 and H42, as well as H3 and H41. The haplotypes from AWWP (H54), KKWR (H56 and H57), and Pakistan (H55) are separated from the nearest Iranian haplotype (H2) by 11 mutations. The structure of the MJ network is intricate, with numerous connections among Iranian haplotypes. H1 and H18 are the most frequent haplotypes. One captive individual from KKWR (JN410341) shares the widespread haplotype H14 from Iran, while another (KU560634) belongs to H18, one of the central haplotypes of Iran, found in Kerman, Hormozgan (mainland and islands), and Sistan and Baluchestan provinces. The animals from AWWP and Pakistan do not share haplotypes with Iranian samples.

**FIGURE 4:**
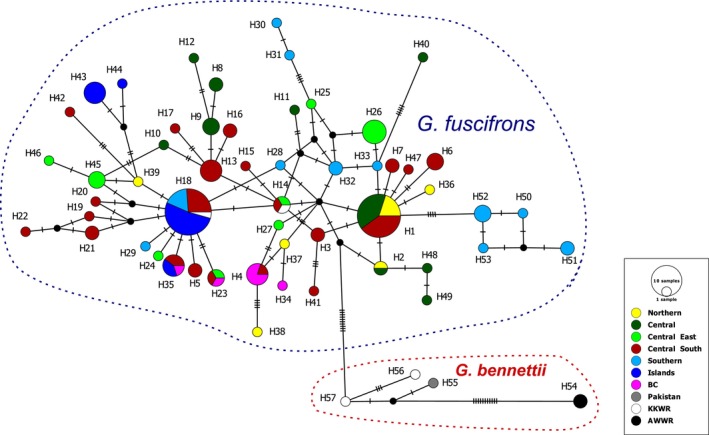
Median‐joining network of cyt *b* sequences of the *G. bennettii* group consisting of *G. fuscifrons* and *G. bennettii* haplotypes (circles). Mutational steps among haplotypes are indicated with dashed lines, and small, filled black circles refer to inferred missing haplotypes. Groups are Northern (1 = Turan NP, 2 = Chah Shirin NHA, 3 = Kavir NP), Central (4 = Abbas Abad WR, 5 = Ardestan, 6 = Siahkoh NP, 7 = Saqand Desert, 8 = Darre Anjir WR, 9 = Bahabad NHA, 10 = Naybandan WR, 11 = Ariz WR, 12 = Bafq PA, 13 = Ravar), Central East (14 = Shile PA, 15 = Rahmatzei BC, 16 = Mokesorkh NHA, 18 = Bolbolab NHA), Central South (17 = Boruyeh WR, 19 = Bahram‐e Goor PA, 20 = Qatruyeh NP, 21 = Sang‐e Mes PA, 22 = Khabr NP, 23 = Kerman, 24 = Tarom PA, 25 = Mosafer Abad Plain, 26 = Bazman NHA, 27 = Hormoud PA), Southern (29 = Geno PA, 31 = Bandar‐e Khamir, 33 = Barkohi Village, 36 = Bandar‐e Lengeh, 37 = Koh‐e Pozak PA, 38 = Sohran Village, 39 = Gando PA, 40 = Chabahar), Islands (30 = Hormoz Island, 32 = Larak Island, 34 = Lavan Island, 35 = Hengam Island), BC (28 = Nayband NP), Pakistan, KKWR, and AWWP. The numbering for each population corresponds to the sorted order based on latitude in Figure 2.

Geographical clustering into ten groups was used for the second MJ network (Figure 4), revealing that the southern islands share haplotypes with the Iranian mainland, except for Lavan Island, which has two unique haplotypes (H43 and H44). In Figure 4, Iranian populations are divided into seven groups based on latitude and longitude, considering geographical distance for populations with the same latitude, except for isolated southern islands (Islands) and Nayband NP, which serves as a breeding center (BC) with individuals from different origins.

### 3.3 Population Differentiation and Expansion

Using the cyt *b* sequences, molecular diversity indices were determined (Table 1) for the *G. bennettii* group (the Iranian and a few Indian/Pakistani individuals), with haplotype diversity (*h*: 0.947 ± 0.011), nucleotide diversity (*π*: 0.00471 ± 0.0004), and the mean number of pairwise differences (*k*: 5.37).

**TABLE 1:** Cyt *b* mtDNA (1140 bp) genetic diversity revealed for proposed species of jebeer and chinkara.

	*n*	*S*	*H*	*h ±* SD	*π ±* SD	*k*
Jebeer (Iran + KKWR)	136	54	53	0.943 ± 0.011	0.00394 ± 0.00023	4.49
Chinkara (KKWR + AWWP)	5	16	4	0.9 ± 0.161	0.00772 ± 0.00175	8.8
*Gazella bennettii* group (all sequences of jebeer and chinkara)	141	76	57	0.947 ± 0.011	0.00471 ± 0.0004	5.37

Abbreviations: *h* = haplotype diversity; *H* = number of haplotypes; *k* = mean number of pairwise differences; *n* = number of individuals; *S* = number of segregating sites; *π* = nucleotide diversity.

**FIGURE 5:**
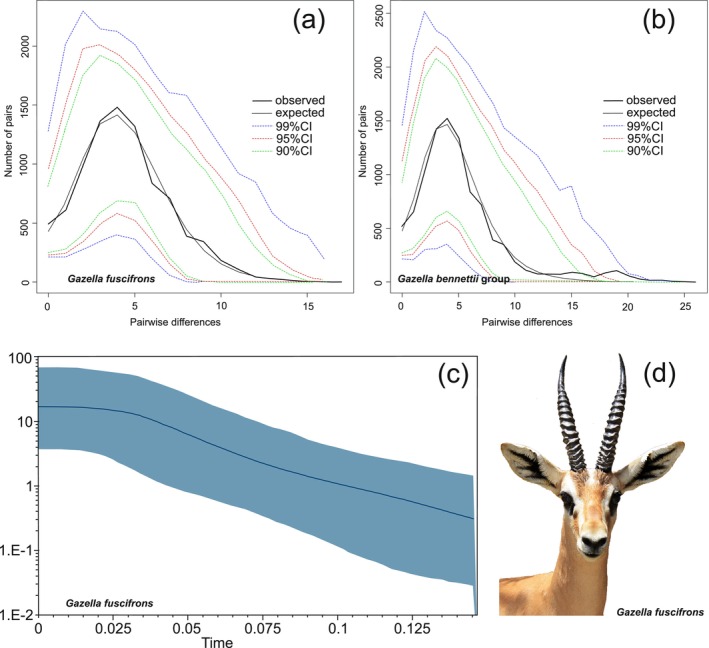
(a) Mismatch distributions for *G. fuscifrons* and (b) the *G. bennettii* group. Depicted are observed (solid black lines) and expected (solid gray lines) frequencies obtained under a model allowing for demographic expansion. (c) Bayesian Skyline Plot of cyt *b* haplotypes of *G. fuscifrons*. (d) Male jebeer from southeastern Iran. In the Bayesian Skyline Plot, the x‐axis represents time in 1000 years ago (kya), the y‐axis shows the effective population size of females (Ne) multiplied by generation time (T), with the solid line representing the median effective population size (NeT) over time to the present, and the blue areas indicating the 95% HPD.

Mismatch distributions were unimodal and consistent with a population expansion for jebeer (Figure 5a) and the *G. bennettii* group (Figure 5b). A similar pattern was observed using *R*_2_, Tajima's *D* (significantly negative), and Fu's *F*_s_ (significantly negative) statistics (Table 2). This pattern was significant for both jebeer and the *G. bennettii* group (*p* < 0.05).

**TABLE 2:** Tests for population expansion for proposed subspecies of *Gazelle bennettii* group using *R*_2_ (Ramos‐Onsins and Rozas 2002), Tajima's *D* (Tajima 1989), and Fu's *F*_s_ (Fu 1997).

	*R*_2_	*p*	Fu's *F*_s_	*p*	Tajima's *D*	*p*
Jebeer	0.0403	**	−43.825	**	−1.73875	*
Chinkara	0.2365	**	1.395	n.s.	1.06914	(n.s.)
*Gazelle bennettii* group (all sequences of jebeer and chinkara)	0.0341	**	−43.804	**	−1.95418	**

Abbreviation: n.s. = not significant.

* Significant (*p* < 0.05).

** Highly significant (*p* < 0.01).

Neutrality tests indicated that jebeers had experienced population expansion in the past (Table 2), and the unimodal mismatch distribution curve further supported historical population expansion in jebeer (Table 2; Figure 5a). Additionally, the observed pattern of BSP suggests that jebeer populations remained demographically stable, followed by population expansion in the last 150 thousand years (Figure 5c).

## 4 Discussion

This study represents the first attempt to use genetic information to assess the potential presence of multiple species within the *G. bennettii* group (Figure 1) and investigate the distinction between Iranian and Indian populations (Figure 3b). Previous phylogenetic studies have been conducted on several gazelle species, but not specifically on the *G. bennettii* group. Cyt *b* datasets have demonstrated sufficient resolution power in diagnosing potential cryptic diversity within gazelles, as evidenced by the separation of *G. marica* from *G. subgutturosa* (Wacher et al. 2011; Fadakar et al. 2019) and *G. gazella* from *G. arabica* (Wronski et al. 2010; Lerp et al. 2013), as well as distinguishing subspecies, such as the goitered gazelle (Fadakar, Malekian, et al. 2020) and the Farur Island subspecies of *G. arabica dareshurii* from Iran (Fadakar et al. 2021).

### 4.1 Phylogeny of the *G. bennettii* Group

In this study, samples from jebeer populations were collected noninvasively across habitats in Iran (Figure 2), covering areas where the putative *G. fuscifrons* and *G. shikarii* species have been described by Groves and Grubb (2011). In the phylogenetic trees (Figures 3 and S1), all jebeer samples are located in a distinct clade, suggesting that there is only one single species (*G. fuscifrons* with priority over *G. shikarii*) throughout Iran based on the cyt *b* dataset. Within the *G. bennettii* group, cyt *b* sequences indicated a clear division into two distinct lineages: the Iranian jebeer and the Indian chinkara. The lack of shared haplotypes, coupled with a number of fixed differences between the two geographical clades, suggests that they may have been isolated for a significant period of time. Cyt *b* sequences from India, Pakistan, and two sequences of unknown origin were assigned to the chinkara clade. However, more sampling from Indian chinkara is required for confirmation.

Nuclear intron markers (CHD2 and ZNF618) exhibited limited variation and did not demonstrate diversity within the *G. bennettii* group (Tables S2 and S3). Nonetheless, these markers were consistent in delineating jebeer from the goitered gazelle, indicating their usefulness for detecting hybridization between jebeer and goitered gazelle, a concern in jebeer breeding centers and areas with overlapping habitats (Fadakar, Bärmann, et al. 2020).

### 4.2 Geographic Patterns of the *G. bennettii* Group

The distinction between the Iranian jebeer and the Indian chinkara suggests that the Indus River may act as a geographical barrier, separating the jebeer (found west of the Indus River) from the chinkara (located east of the Indus River) (see Figure 3b). To our knowledge, there are currently no studies assessing genetic variation in vertebrate taxa on the eastern and western sides of the river. However, mtDNA control region sequences of the golden jackal ( *Canis aureus* ) indicate that Indian and Eurasian haplotypes are nearly exclusive to each side of the river (Milanlou et al. 2024).

Differences among species or subspecies in the magnitude of divergence may also reflect varying levels of gene flow across the river, irrespective of how the subspecies initially became separated. This phenomenon is related to the species‐specific dispersal abilities and ecological requirements of each taxon (Smith et al. 2014; Harvey et al. 2017; Naka and Brumfield 2018; Lavinia et al. 2019). Therefore, it is possible that the ancestral populations of the *G. bennettii* group were divided by the river through a vicariant event. However, the differing timings of divergence among the species may suggest that population differentiation occurred after the establishment of the river axis, likely due to dispersal from one side to the opposite margin. This mechanism is also observed in other riverine barriers, such as the Amazon (Smith et al. 2014; Naka and Brumfield 2018).

Due to the lack of genetic data from Pakistan and Afghanistan, there is currently no information available comparing the regions east and west of the Indus River. Consequently, this article represents the first examination of a desert ungulate with a wide distribution range in this region. In this regard, the two separate clades of COI sequences (PP = 1) (Figure S2) from the western and eastern regions of the Indus River align with the separation created by the Indus River barrier. However, further research is necessary, including the collection of samples from both sides of the Indus River.

### 4.3 Divergence Timing

Results showed that jebeer (*G. fuscifrons*) and chinkara ( *G. bennettii* ) as two main clades within the *G. bennettii* group, diverged 0.895 Mya in the middle Pleistocene (Figure S3). This divergence is potentially related to climate changes during the Early–Middle Pleistocene transition (1.4–0.4 Mya) (Head and Gibbard 2015), characterized by glacial–interglacial cycles (Clark et al. 2006; Willeit et al. 2019; de Jong et al. 2020). These climate changes would have facilitated distribution and range expansion into India, and a major intensification of the Asian monsoon system (Peng et al. 2020) may have led to the Indus River acting as a barriers between them, resulting in vicariant speciation.

### 4.4 Connectivity of Jebeer in Iran

Previous studies have reported seasonal migrations of goitered gazelles in herds exceeding 100 individuals (Esfahani and Karami 2005; Khosravi et al. 2018). In contrast, jebeer populations typically consist of 3–5 individuals or solitary animals, distributed across regions with smaller populations (usually fewer than 50 individuals per area). Morphological differences have been observed between northern and southern jebeer populations (Karami et al. 2002), such as those in Kavir NP in the north and the Sistan and Baluchestan populations in southeastern Iran (Figure 5d). This has led to the presumption that only goitered gazelles are migratory, while jebeers are not.

The absence of migration or movement between jebeer populations may result in the formation of distinct groups. However, analysis of haplotypes reveals a complex network of connections among jebeer populations in Iran, indicating that these populations are interconnected rather than forming distinct groups based on the cyt *b* dataset (Figure 4). A possible explanation for this haplotype connectivity is that the subdivision among Iranian geographical populations occurred relatively recently, following the Last Glacial Maximum (LGM). The cyt *b* gene lacks sufficient resolution to detect any subdivision within this species. Consequently, even if the different geographical populations have been isolated for some time and have developed unique morphological traits, the cyt *b* data do not possess the sensitivity required to identify such patterns. Furthermore, it is evident that cyt *b* can only reveal demographic changes—specifically expansions—of the species from a relatively long time ago, whereas the species has been experiencing a decline in more recent times (Akbari 2014). In this context, utilizing genomic or subgenomic datasets, as well as microsatellite data, is recommended to uncover more recent variations across both large and small geographic scales.

### 4.5 Jebeer Populations on the Persian Gulf Islands

Jebeer populations have been identified on four islands: Hormoz (H18 and H35), Hengam (H18), Larak (H18), and Lavan (H43 and H44). The first three islands are in close proximity to one another and share haplotypes with the mainland, while Lavan exhibits unique haplotypes (Figure 2). We speculate that a similar scenario of island isolation occured in the Persian Gulf after the LGM, due to rising sea levels, as observed on Farur Island (Fadakar et al. 2021), for these islands. According to this hypothesis, the Persian Gulf functioned as a river valley with a few hilly outcrops during the LGM (Kennett and Kennett 2007), and the islands subsequently emerged as a result of sea‐level changes and the gradual flooding of the dry gulf basin from the Strait of Hormuz (Lambeck 1996; Kennett and Kennett 2007).

The sharing of haplotypes with the mainland is valuable for studying the potential of island populations in reintroduction and reinforcement programs (Lerp et al. 2014), while the presence of unique haplotypes is important for the conservation of local diversity and adaptations (Fadakar et al. 2021) make these southern islands valuable resources for conservation efforts and further research.

### 4.6 Study Limitations

Although our data revealed that the *Gazelle bennettii* group has diverged into two distinct lineages—the Iranian jebeer (*G. fuscifrons*) and the Indian chinkara ( *G. bennettii* ) clades—this separation is supported solely by preliminary mtDNA analysis. The nuclear markers employed do not demonstrate sufficient variation to address questions concerning the taxonomic status of *G. bennettii* .

Most of the samples in this study were collected from various regions of Iran; however, the availability of suitable samples for DNA extraction from Pakistan and India was limited. The sample size from the eastern side of the Indus River was relatively small, which may have hindered the detection of subtle differences and affected statistical significance. The phylogeographic analyses of the *G. bennettii* group would be more comprehensive if additional samples from India and Pakistan were included.

Mitochondrial DNA alone is insufficient as the exclusive source of species‐defining data due to several limitations, including reduced effective population size, introgression, maternal inheritance, recombination, inconsistent mutation rates, heteroplasmy, and complex evolutionary processes (Kowalczyk et al. 2021). Consequently, this study serves as a baseline assessment for future evaluations and for testing the hypothesis that the Indus River may function as a geographical barrier between the jebeer (west of the Indus River) and the chinkara (east of the Indus River).

### 4.7 Conservation Implications

Our study indicates that the jebeer represents a distinct lineage, separate from the non‐endangered chinkara found in India. This finding is highly relevant to the IUCN Red List assessment of the jebeer. Currently, the chinkara is classified as least concern (LC); however, the Iranian population of chinkara (jebeer) is assessed at the national level as endangered (EN) (Yusefi et al. 2019). Therefore, grouping the jebeer with the Indian population under the same global assessment underestimates its true endangered status.

In Iran, the haplotype distribution map of the jebeer can provide a fundamental genetic framework to guide conservation efforts across Iranian protected areas and captive breeding centers. Consequently, any future management plans should be based on this new finding and treat these two lineages separately.

## Author Contributions

**Davoud Fadakar:** conceptualization (equal), data curation (lead), formal analysis (lead), investigation (lead), methodology (equal), visualization (lead), writing – original draft (lead), writing – review and editing (equal). **Mansoureh Malekian:** conceptualization (equal), supervision (equal), writing – original draft (equal), writing – review and editing (equal). **Mahmoud Reza Hemami:** supervision (equal), writing – review and editing (equal). **Hamid Reza Rezaei:** investigation (equal), resources (equal), writing – review and editing (equal). **Hannes Lerp:** resources (equal), validation (equal), writing – review and editing (equal). **Eva V. Bärmann:** methodology (equal), validation (equal), writing – review and editing (equal).

## Conflicts of Interest

The authors declare no conflicts of interest.

## Supporting information


**FIGURE S1.** (a) Phylogeny of jebeer (blue), chinkara (red), and goitered gazelle × jebeer hybrids (green) from Bayesian analysis of cyt *b* gene sequences with partitioning scheme. (b) Bayesian analysis based on the 2506 bp from concatenated analysis of cyt *b* and two nuclear introns (CHD2 and ZNF618) with partitioning scheme. The trees were summarized with the majority‐rule consensus tree. Numbers above branches are posterior probabilities.

**FIGURE S2.** Phylogeny of sequences from the western (blue, *G. fuscifrons*) and eastern parts of the Indus River (red, *G. bennettii* ) from Bayesian analysis of COI gene sequences.

**FIGURE S3.** Bayesian phylogenetic tree of *G. fuscifrons* (blue), *G. bennettii* (red), and goitered gazelle × jebeer hybrids (green) indicating divergence time (in Mya) estimates based on the mtDNA cyt *b*.

**FIGURE S4.** Median‐joining network of cyt *b* sequences of *G. bennettii* group consisting of *G. fuscifrons* and *G. bennettii* haplotypes. Mutational steps among haplotypes are signaled with dash lines and small, filled black, circles refer to inferred missing haplotypes. Each circle represents a different haplotype, whereby areas of circles are proportional to the number of sampled individuals (see the legend for the circle sizes of one and ten samples).

**TABLE S1.** List of cyt *b* and nuclear introns (ZNF618 and CHD2) sequences of *G. fuscifrons* and *G. bennettii* , *G. subgutturosa* , and *G. subgutturosa* × *G. fuscifrons* hybrid samples used in this study, downloaded from GenBank or obtained by the authors of this study.

**TABLE S2.** Variable sites in the intron sequence of ZNF618.

**TABLE S3.** Variable sites in the intron sequence of CHD2.

## Data Availability

DNA sequences have been deposited in GenBank under the accession no: PQ809265–PQ809448.
