# Supplementary figures and images for: Phylogenetic Assessment of Gazella bennettii: A Genetic Framework for the Conservation of the Endangered Jebeer in Iran
Source: Ecol Evol. 2025 Feb 12;15(2):e70954. doi: 10.1002/ece3.70954 (PMC11821286; doi:10.1002/ece3.70954)

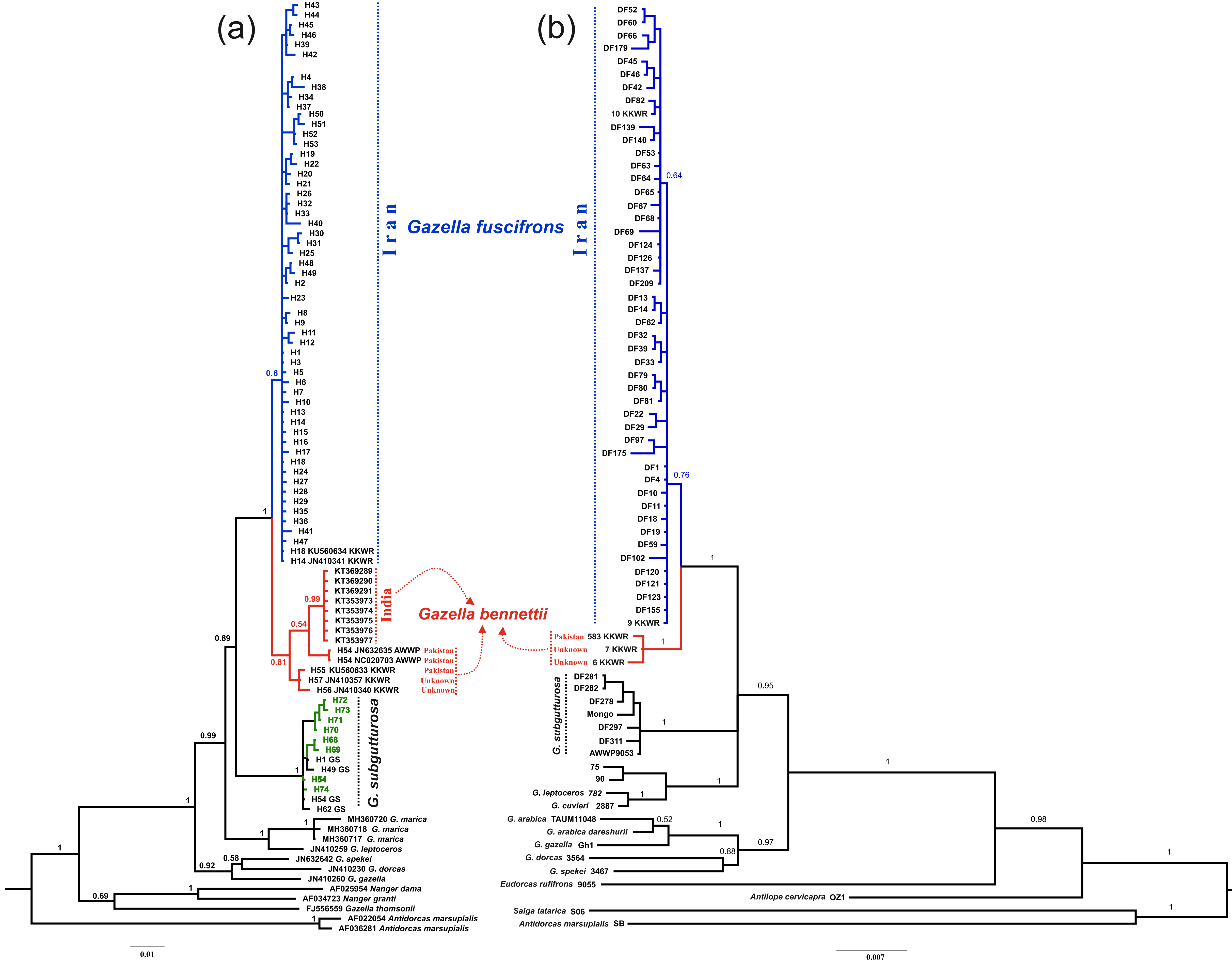

Supplement: Supplementary file 1 — FIGURE S1. (a) Phylogeny of jebeer (blue), chinkara (red), and goitered gazelle × jebeer hybrids (green) from Bayesian analysis of cyt b gene sequences with partitioning scheme. (b) Bayesian analysis based on the 2506 bp from concatenated analysis of cyt b and two nuclear introns (CHD2 and ZNF618) with partitioning scheme. The trees were summarized with the majority‐rule consensus tree. Numbers above branches are posterior probabilities. [file ECE3-15-e70954-s007.pdf]

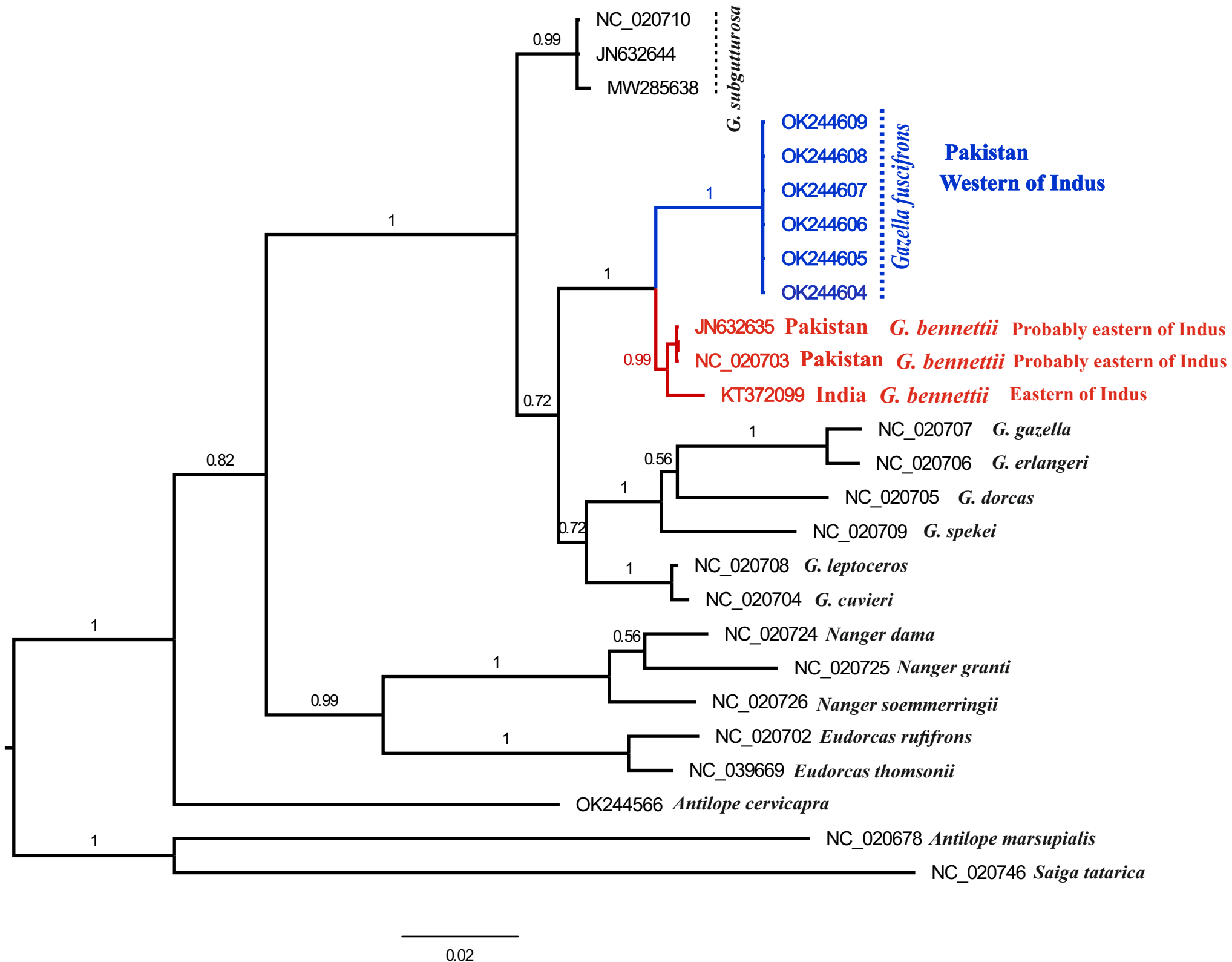

Supplement: Supplementary file 2 — FIGURE S2. Phylogeny of sequences from the western (blue, G. fuscifrons) and eastern parts of the Indus River (red, G. bennettii ) from Bayesian analysis of COI gene sequences. [file ECE3-15-e70954-s002.pdf]

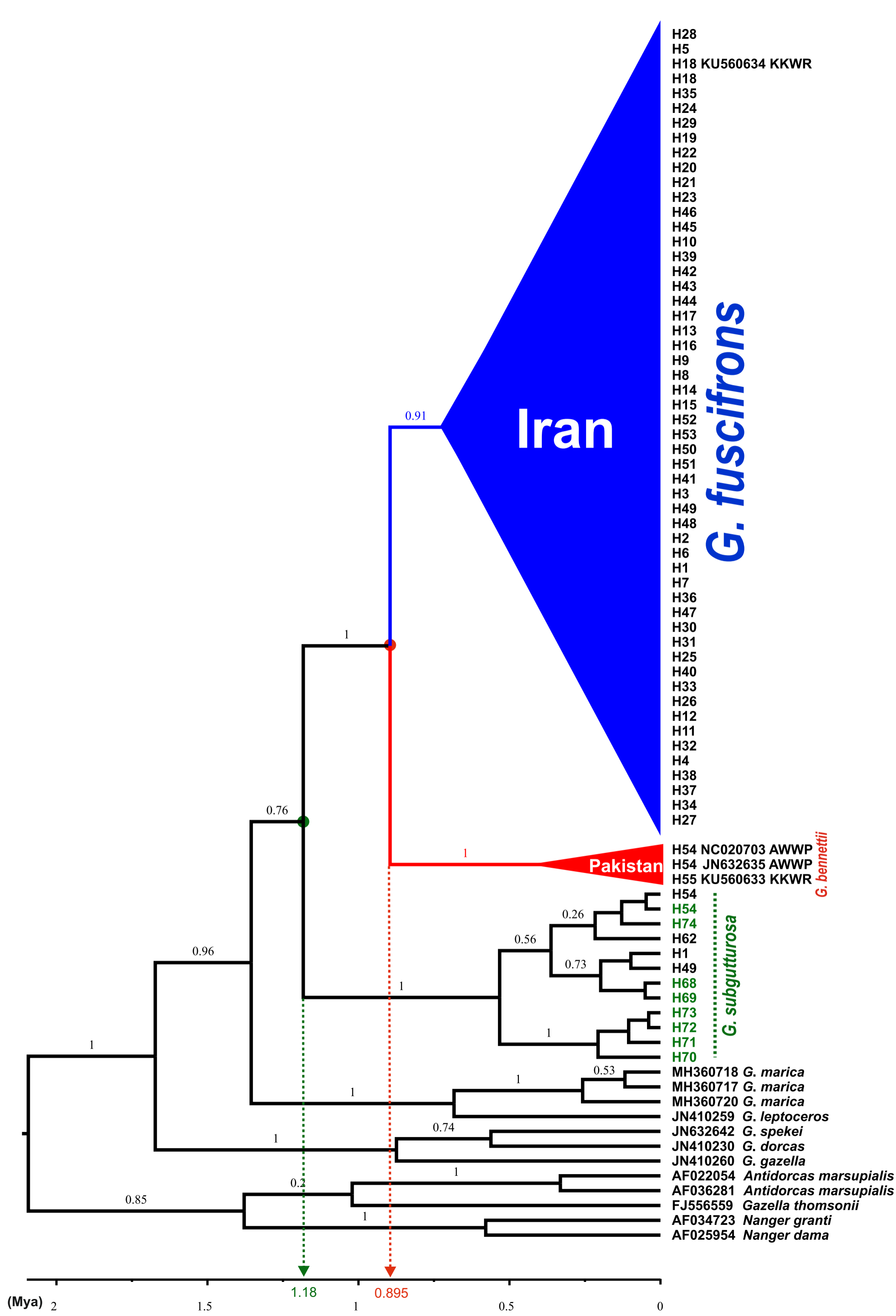

Supplement: Supplementary file 3 — FIGURE S3. Bayesian phylogenetic tree of G. fuscifrons (blue), G. bennettii (red), and goitered gazelle × jebeer hybrids (green) indicating divergence time (in Mya) estimates based on the mtDNA cyt b. [file ECE3-15-e70954-s004.pdf]

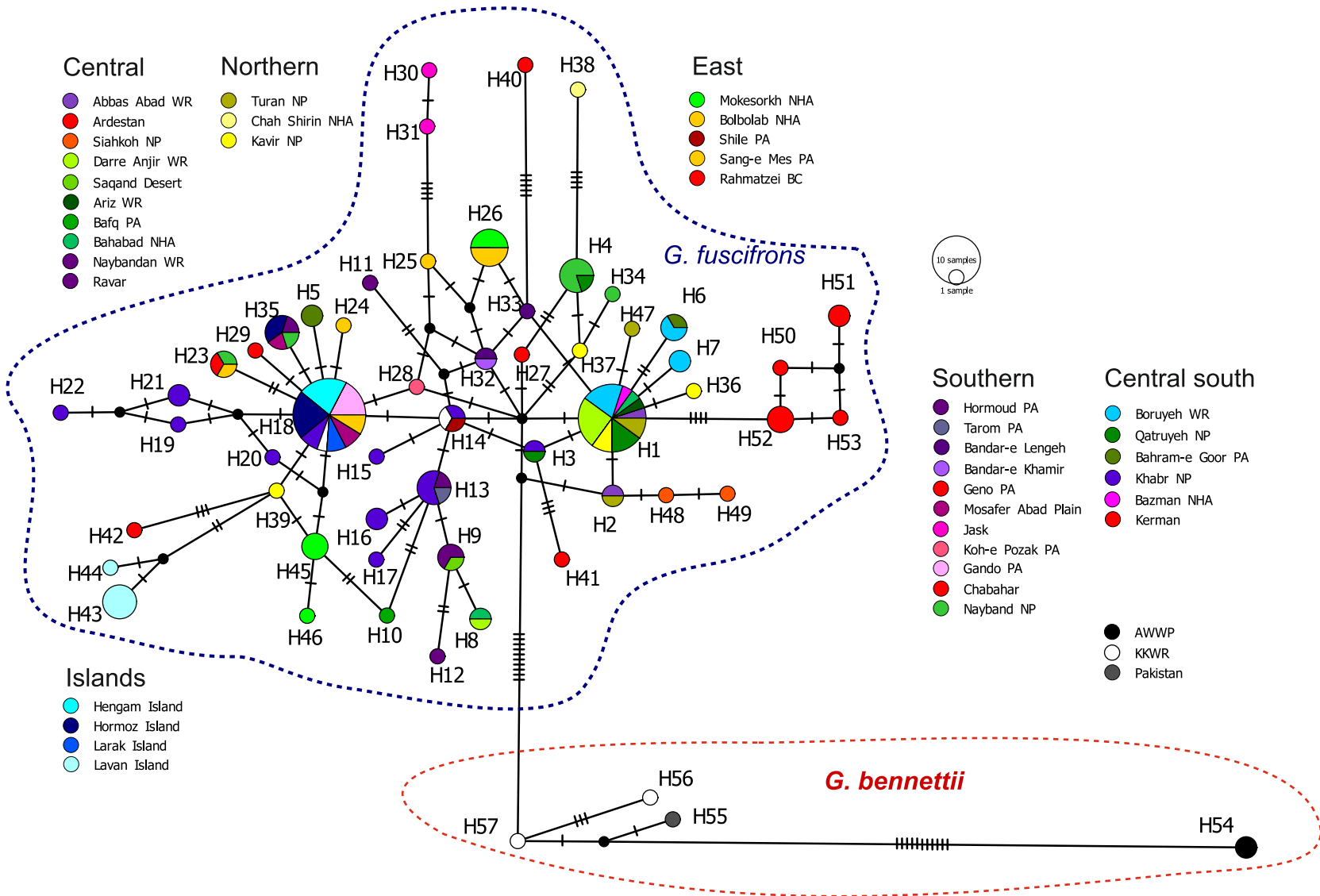

Supplement: Supplementary file 4 — FIGURE S4. Median‐joining network of cyt b sequences of G. bennettii group consisting of G. fuscifrons and G. bennettii haplotypes. Mutational steps among haplotypes are signaled with dash lines and small, filled black, circles refer to inferred missing haplotypes. Each circle represents a different haplotype, whereby areas of circles are proportional to the number of sampled individuals (see the legend for the circle sizes of one and ten samples). [file ECE3-15-e70954-s006.pdf]
