# Supplementary material for: Phylogenetic Assessment of Gazella bennettii: A Genetic Framework for the Conservation of the Endangered Jebeer in Iran
Source: Ecol Evol. 2025 Feb 12;15(2):e70954. doi: 10.1002/ece3.70954 (PMC11821286; doi:10.1002/ece3.70954)
Supplement: Supplementary file 6 — TABLE S2. Variable sites in the intron sequence of ZNF618. [file ECE3-15-e70954-s003.docx]

| Table S2. Variable sites in the intron sequence of ZNF618. | | | | | | | | | | | | | | | | | | | | | | | | |  |
| --- | --- | --- | --- | --- | --- | --- | --- | --- | --- | --- | --- | --- | --- | --- | --- | --- | --- | --- | --- | --- | --- | --- | --- | --- | --- |
| Species | Location | ID | Variable sites | | | |  |  | Insertion |  | Variable sites | | |  |  |  |  |  |  |  |  |  |  |  | |
|  |  |  | 84 | 108 | 122 | 136 | 189 |  | 213-218 |  | 238 | 275 | 278 | 305 | 326 | 355 | 356 | 398 | 460 | 501 | 607 | 627 | 641 | 666 | |
| *G. fuscifrons* | Iran | TRAN2 | G | C | C | A | C |  | – |  | C | C | C | G | G | G | G | G | A | C | C | – | – | – | |
|  |  | DNJR1 | . | . | . | . | T |  | – |  | T | . | . | . | . | . | A | . | . | . | ? | – | – | – | |
|  |  | LVN1 | . | . | . | . | T |  | – |  | T | . | . | . | . | . | . | . | . | . | . | – | – | – | |
|  |  | BKLT2 | . | . | . | . | T |  | – |  | T | . | T | . | . | . | . | . | . | . | . | – | – | – | |
|  |  | BGOR1 | . | . | T | C | . |  | – |  | . | . | . | A | . | . | . | . | . | . | . | – | – | – | |
|  |  | HRMD1 | . | . | . | . | . |  | – |  | . | . | . | . | . | . | . | . | C | . | . | – | – | – | |
|  |  | HRMD5 | . | . | . | . | . |  | – |  | . | . | . | R | . | R | . | . | . | Y | . | – | – | – | |
|  |  | TRAN3, BKLT3, BLNG2, BGOR3 | . | . | . | . | . |  | – |  | . | . | . | . | . | . | . | . | . | . | . | – | – | – | |
| *G. bennettii* | Pakistan | 583 | R | . | . | . | . |  | – |  | . | . | . | . | . | . | . | . | . | . | Y | – | – | – | |
|  | KKWR, Unknown | 6 | R | . | . | . | . |  | – |  | . | . | . | . | . | . | . | . | . | . | . | G | G | – | |
| *G. fuscifrons* | Iran | BIAZE1, ARIZ1, KHBR1, KHBR8, POZK1, | . | . | . | . | . |  | CAGGGT |  | . | . | . | . | . | . | . | . | . | . | . | – | – | – | |
|  |  | CBHR1, JASK1, BLNG1, KVIR3, GENO1, |  |  |  |  |  |  |  |  |  |  |  |  |  |  |  |  |  |  |  |  |  |  |  |
|  |  | BOR1, BOR2, MSFR2, HRMZ1, BGOR2, |  |  |  |  |  |  |  |  |  |  |  |  |  |  |  |  |  |  |  |  |  |  |  |
|  |  | MOKE4, HNGM4, TARM4 |  |  |  |  |  |  |  |  |  |  |  |  |  |  |  |  |  |  |  |  |  |  |  |
|  |  | DNJR6, NBND1, NBND8, NIBN1 | . | . | . | . | . |  | CAGGGT |  | . | T | . | . | . | . | . | . | . | . | . | – | – | – | |
|  |  | SHLE1, IRSH1 | . | . | . | . | T |  | CAGGGT |  | . | . | . | . | . | . | . | . | . | . | . | – | – | – | |
|  |  | DNJR2 | . | . | . | . | T |  | CAGGGT |  | T | . | . | . | . | . | . | . | . | . | . | – | – | – | |
|  |  | RMZE1 | . | . | . | . | . |  | CAGGGT |  | T | . | . | . | . | . | . | . | . | . | . | – | – | – | |
|  |  | BGOR4 | . | . | . | T | . |  | CAGGGT |  | . | . | . | A | . | . | . | . | . | . | . | – | – | – | |
|  |  | SMES3, BOR5 | . | . | . | . | . |  | CAGGGT |  | . | Y | . | . | . | . | . | . | . | . | . | – | – | – | |
|  |  | HRMD4 | ? | ? | ? | ? | ? |  | ? |  | Y | Y | . | A | . | . | . | . | . | . | . | – | – | – | |
|  |  | KHBR14 | ? | ? | . | ? | ? |  | CAGGGT |  | T | . | . | . | . | . | . | . | . | . | ? | – | – | – | |
|  |  | KHBR15 | ? | ? | ? | ? | . |  | CAGGGT |  | . | . | . | . | . | . | . | . | . | ? | ? | – | – | – | |
|  |  | BGOR5 | ? | ? | ? | ? | ? |  | ? |  | Y | . | . | . | . | . | . | . | . | . | . | – | – | – | |
|  |  | BGOR6 | ? | ? | ? | ? | ? |  | ? |  | . | . | . | . | . | . | . | . | . | . | . | – | – | – | |
|  |  | CHAH1 | ? | ? | ? | ? | ? |  | CAGGGT |  | . | . | . | . | . | . | . | . | . | . | . | – | – | – | |
|  |  | 10 | . | . | . | . | . |  | CAGGGT |  | . | T | . | . | . | . | . | . | . | . | . | – | – | ? | |
|  |  | 9 | . | . | . | . | . |  | CAGGGT |  | . | . | . | . | ? | ? | ? | ? | ? | ? | ? | ? | ? | ? | |
| *G. bennettii* | KKWR, Unknown | 7 | . | . | . | . | . |  | CAGGGT |  | . | . | . | . | ? | . | . | R | . | . | . | – | – | T | |
