# Supplementary material for: Phylogenetic Assessment of Gazella bennettii: A Genetic Framework for the Conservation of the Endangered Jebeer in Iran
Source: Ecol Evol. 2025 Feb 12;15(2):e70954. doi: 10.1002/ece3.70954 (PMC11821286; doi:10.1002/ece3.70954)
Supplement: Supplementary file 7 — TABLE S3. Variable sites in the intron sequence of CHD2. [file ECE3-15-e70954-s001.docx]

| Table S3. Variable sites in the intron sequence of CHD2. | | | | | | |
| --- | --- | --- | --- | --- | --- | --- |
| Species | ID | Variable sites | | | | |
|  |  | 109 | 216 | 221 | 236 | 273 |
| *G. fuscifrons* | All other | C | C | T | C | T |
| *G. bennettii* | 583, 7, 6 | . | . | . | . | . |
| *G. fuscifrons* | BKLT3 | T | T | . | . | . |
| *G. fuscifrons* | BGOR3, HRMD5 | . | . | C | . | . |
| *G. fuscifrons* | 9 | . | . | . | Y | Y |
